# Model-Based Design of Tree WSNs for Decentralized Detection †

**DOI:** 10.3390/s150820608

**Published:** 2015-08-20

**Authors:** Ashraf Tantawy, Xenofon Koutsoukos, Gautam Biswas

**Affiliations:** 1 RasGas Company Limited, Doha 24200, Qatar; 2 Institute for Software Integrated Systems, Department of Electrical Engineering and Computer Science, Vanderbilt University, Nashville, TN 37235, USA; E-Mails: xenofon.koutsoukos@vanderbilt.edu (X.K.); gautam.biswas@vanderbilt.edu (G.B.)

**Keywords:** cyber-physical systems, decentralized detection, tree sensor networks, networked information fusion, wireless sensor network, multiaccess communication, transmission control policy, modeling, simulation, optimization

## Abstract

The classical decentralized detection problem of finding the optimal decision rules at the sensor and fusion center, as well as variants that introduce physical channel impairments have been studied extensively in the literature. The deployment of WSNs in decentralized detection applications brings new challenges to the field. Protocols for different communication layers have to be co-designed to optimize the detection performance. In this paper, we consider the communication network design problem for a tree WSN. We pursue a system-level approach where a complete model for the system is developed that captures the interactions between different layers, as well as different sensor quality measures. For network optimization, we propose a hierarchical optimization algorithm that lends itself to the tree structure, requiring only local network information. The proposed design approach shows superior performance over several contentionless and contention-based network design approaches.

## 1. Introduction

The deployment of wireless sensor networks (WSNs) in decentralized detection applications is motivated by the availability of low cost sensors, combined with the advances in communication network technologies. In decentralized detection (DD), multiple sensors collaborate to distinguish between two or more hypotheses. In many practical applications, sensors are distributed geographically and connected in a tree configuration for energy efficiency. Each sensor task is to sample the environment, pre-process the data, and communicate the information to the fusion center for final decision-making.

The recent use of WSNs in decentralized detection applications brings new challenges to the design of sensor networks. Protocols for communication layers have to be co-designed to optimize the detection performance. The layered approach commonly adopted to design wireless networks may not be appropriate for detection applications, as it neither provides the optimal resource allocation nor exploits the application domain knowledge. A cross-layer design approach is desired for an efficient implementation of WSNs in decentralized detection applications.

In this paper, we pursue a cross-layer approach to design a tree WSN for detection applications. We attempt to strike a balance between theoretical decentralized detection work and practical sensor networks. To achieve this goal, we make the following design assumptions: (1) The minimal movement of sensor nodes: This assumption allows us to consider the large-scale fading component only for the physical channel, hence simplifying the analysis. (2) Slotted ALOHA MAC: The traditional assumption of a dedicated orthogonal channel between each sensor node and its parent node may not be feasible in practice. The Slotted ALOHA multiaccess scheme, on the other hand, has been successfully deployed in practice. We use a simplified version of the slotted ALOHA protocol, ignoring the protocol specifics, to keep the analysis tractable. (3) Synchronization: We assume that sensors are synchronized, which allows us to model the network as a discrete time system, hence simplifying the analysis. (4) Transmission scheme: We assume two transmission schemes, direct transmission where raw observations are sent directly to the fusion center without local processing and in-network processing where information is compressed and quantized locally before transmission. (5) Suboptimal solution: Practical WSNs require solutions for optimization problems that could be executed in real time, while being scalable with network size. We resort to a suboptimal, yet efficient solution as opposed to integer programming problems that arise when trying to use a deterministic transmission scheme.

The rest of the paper is organized as follows: Section 2 summarizes the related work. Section 3 presents the problem formulation. Section 4 explains the system model. Section 5 presents the solution of the optimization problem to obtain the optimal network design parameters. Section 6 presents the performance evaluation for the proposed design, in comparison to several contentionless and contention-based network design schemes, using a numerical example. The work is concluded in Section 7.

## 2. Related Work

The classical problem for decentralized detection that has been extensively addressed is the design of signal processing algorithms at the application layer for different network topologies [1]. Several variations have been introduced to the original problem to account for various network resource constraints [2,3,4]. Such variations are still recently addressed. For example, the value of feedback messages from the fusion center to sensor nodes for single hop networks is studied in [5]. The distributed detection problem for balanced binary relay trees, where nodes and links fail with certain probabilities, is considered in [6].

The cross-layer design approach has been recently explored for the design of media access control (MAC) protocols for parallel topology (direct transmission) sensor networks in detection applications. Decision fusion over slotted ALOHA MAC employing a collision resolution algorithm is studied in [7]. A thorough investigation of the design of MAC transmission policies to minimize the error probability has been considered in [8], where sensors are assumed non-identical and the MAC policy is assumed stochastic. The cross-layer approach is also considered in [9], where an integrated model for the physical channel and the queuing behavior for sensors is developed.

For tree networks, energy-efficient routing for signal detection in WSNs is considered in [10], where the objective is to find the optimal route for local data from a target location to the fusion center, in order to maximize the detection performance or to minimize the energy consumption. Cooperative routing for distributed detection in large sensor networks is studied in [11] using a link metric that characterizes the detection error exponent. Optimal communication rate allocation for multihop sensor networks deployed for DD is studied in [12], where no medium access contention is assumed. For a survey on the interplay between signal processing and networking in sensor networks, see [13] and the references therein.

Our work is different in three main aspects: (1) we integrate the physical layer, MAC layer and the detection application layer in one unified system model; (2) we include the three quality measures that were previously treated separately, namely the quality of information (QoI), channel state information (CSI) and residual energy information (REI) for each sensor; and (3) we assume sensor networks with a finite number of sensors, in contrast to the infinite number of sensors assumption typically used in asymptotic analysis. We design the optimal transmission control policy (XCP) that coordinates the communication between sensor nodes connected in a tree configuration. Our approach formulates the detection performance measure as a function of the parameters of the integrated system model. We then solve a constrained optimization problem to obtain the XCP variables that maximize the detection performance.

We summarize the contributions of our work, as compared to existing literature, in the following main points: (1) Integrated model for the detection system: The model captures the physical channel, MAC protocol and the detection application models and their interactions. The model also incorporates the QoI, CSI and REI measures for each sensor. (2) The design of a complete transmission control policy: We design the XCP for the tree topology for a finite number of sensors, rather than asymptotically. The XCP variables include retransmission probabilities and communication rates for all sensor nodes. (3) Enhanced detection performance: We show that the proposed design approach has a significant improvement in the detection performance over several contentionless and contention-based network design approaches. (4) Comparison between direct transmission and in-network processing schemes: We study the design problem when local observations are quantized and show the conditions under which the in-network processing scheme outperforms the direct transmission scheme.

The work presented in this paper represents a generalization of our work in [14] in two main aspects: (1) the single-hop network is generalized to the tree network, and consequently, the optimization problem of the single hop network is a special case of the optimization problem presented in this paper; and (2) in-network processing of information at sensor nodes, which becomes much more important for tree networks, is considered along with the direct transmission scheme considered in [14].

## 3. Problem Formulation

**Figure 1:**
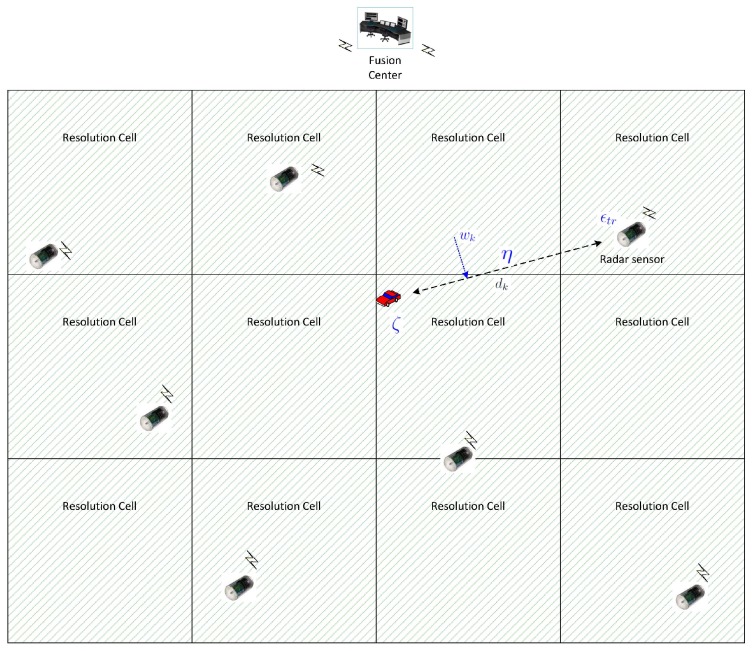
Decentralized detection for a surveillance area with randomly-placed radar-like sensors.

We consider a detection application where a set of sensors are randomly placed to detect the presence of a target, e.g., an intruder or vehicle, in a surveillance area, under the control of a fusion center denoted by FC. The surveillance area is divided into a number of resolution cells that are probed by the local sensors on demand from the fusion center, as depicted in Figure 1. Upon receiving a command from the fusion center to probe a specific resolution cell, local sensors illuminate the resolution cell and sample the reflected signal. The sampled observations are transmitted back to the fusion center for decision making about the presence of a target in the designated resolution cell. The fusion center solves the binary hypothesis testing problem by performing a statistical test on the received observations.

**Figure 2:**
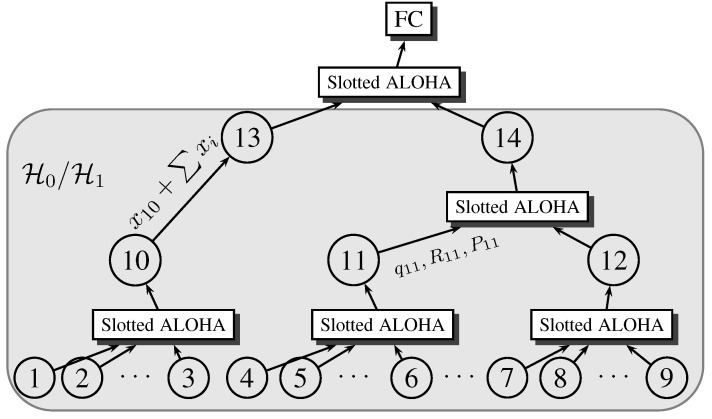
Detection architecture for tree-topology WSN.

We assume fixed local sensors and the fusion center, arranged in a tree structure, as depicted in Figure 2, where the tree is assumed pre-specified, possibly based on sensor locations. Given this detection architecture, it may not be efficient for each local sensor to participate in the surveillance of each resolution cell, as requested by the fusion center. This may be due to one or more of several reasons: (1) the sensor may be far from the resolution cell, making its observations less reliable; (2) the communication channel between the sensor and the fusion center may be unreliable at the time of detection, making information loss more probable; and (3) the sensor may not have enough stored energy to illuminate the designated resolution cell or to send the information to the fusion center. Accordingly, in order to optimize the detection performance without wasting network resources, an XCP needs to be developed. For each resolution cell, the XCP defines the specific sensors, the energy resources, and the network parameters that should be used in each surveillance task. These calculations are based on several factors: (1) the sensor location with respect to the resolution cell; (2) the communication channel state between the sensor and the fusion center; and (3) the energy reserve for the sensor. This information could be sent to the fusion center either periodically or on-demand to recalculate the XCP. The calculated XCP remains valid as long as the sensor attributes previously submitted did not change. We denote that by handshaking overhead, since it does not contribute to the detection task. In this paper, we ignore the handshaking overhead in the development of the system model. However, this overhead may become significant if the environment dynamics are fast. In Section 5.3, we develop an upper bound on the environment dynamics, such that ignoring the handshaking overhead does not affect the model accuracy.

Two approaches are possible to calculate the optimal transmission control policy: (1) the global approach, where the fusion center receives the information from all sensors (through their respective parents), calculates the optimal transmission control policy for each sensor by solving a constrained nonlinear optimization problem and transmits the values of the XCP variables back to the relevant sensors; and (2) the local approach, where each parent node solves a smaller local optimization problem to specify the locally-optimal XCP variables for its child nodes. The global approach may not be feasible in large sensor networks, as it is not scalable with the network size. In addition, the design parameters have to be propagated back from the fusion center to all network nodes. For large sensor networks, the local approach is more practical.

After each sensor receives the optimal values of its XCP variables, the detection process proceeds as follows: The fusion center broadcasts a message to initiate a detection cycle at the local wireless sensors. Each local sensor samples the environment by collecting a number of observations and then forms a data packet and communicates its message to the parent node over a shared wireless link using the slotted ALOHA multiaccess control scheme. Parent nodes relay the information of the child nodes, in addition to their own information, through the tree network until reaching the fusion center. Finally, the fusion center makes a final decision after a fixed amount of time representing the maximum allowed delay for detection.

In this paper, we consider two transmission schemes. (1) In direct transmission, each sensor transmits its raw observations without quantization to the fusion center. Obviously, quantization is necessary for digital communication, but the number of quantization bits is assumed large in this case so that the quantization effect is negligible. Transmission of raw observations guarantees no loss of detection performance at the fusion center. On the down side, observations build-up and accumulate through the tree network. Therefore, the communication rate at relay nodes up in the tree hierarchy has to increase to cope with the volume of data coming from child nodes. This causes a higher probability of information loss due to the high communication rate. (2) In in-network processing, information is compressed by calculating its log likelihood ratio (LLR), then the LLR is quantized before transmission using a limited number of quantization bits. This scheme reduces the communication rate and increases the probability of successful transmission, but suffers from irrecoverable loss of information caused by the in-network processing. We assume uniform quantization to simplify the analysis, as the problem of finding the optimal quantization thresholds for detection applications have proven to be very difficult, even with small network sizes [15].

## 4. System Model

**Figure 3:**
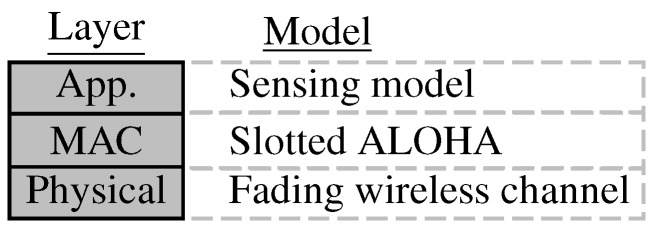
A layered approach to detection system modeling.

In this section, we develop a mathematical model for the detection system depicted in Figure 1 and Figure 2. Our main objective is to relate the detection performance measure to the system design variables. Mathematically, our objective is to obtain an optimization problem of the form:

(1) $$\begin{array}{c} \max_{x}J(x)\\ \text{subject}\;\text{to}\;g_{i}\left(x\right)\geq 0\;\text{for}\;i=1,\ldots ,m \end{array}$$

where *J* represents the performance measure we seek to maximize, x is a vector of decision variables, *i.e.*, the XCP variables in our design problem, and $g_{i}\left(.\right)$ are a set of constraints on the design variables. We follow a layered approach to model the detection system, building the model from the ground up. In each of the subsequent subsections, we model one layer at a time, trying to relate the system variables in this specific layer to the variables in other layers, with the goal of reaching the formulation in Equation (1). Specifically, we follow the layered architecture representation for the system depicted in Figure 3. The physical layer represents the wireless channel model. The MAC layer represents the slotted ALOHA protocol model. Finally, the application layer represents the sensing model and defines the model of the observations obtained by local sensors.

### 4.1. Wireless Channel Model

**Figure 4:**
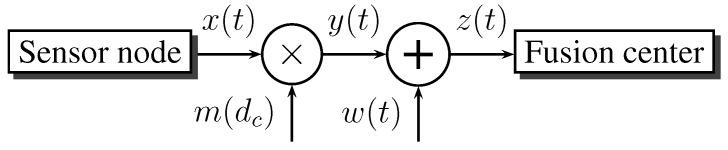
Block diagram for the wireless communication channel.

We present a model for the wireless channel between each parent-child pair in the tree detection network. We derive an expression for the probabilistic channel state in terms of channel, as well as communication parameters. We focus on the case where the sensor nodes and the fusion center have minimal movement and the environment changes slowly. Accordingly, only the slow fading component of the wireless channel is considered. Figure 4 shows the fading channel model, where w(t) is an AWGN with PSD $N_{0}/2$ , and $m(d_{c})$ is the mean path attenuation for a sensor node at a distance $d_{c}$ from the fusion center. Using the Hata path-loss model, the total dB power loss is given by [16]:

(2) $$\begin{array}{cc} P_{L} & =\underset{\mu_{c}}{\underset{︸}{20\log\left(4\pi d_{0}/\lambda_{p}\right)+10\rho_{c}\log\left(d_{c}/d_{0}\right)}}+X_{\sigma_{c}}\;\text{dB} \end{array}$$

where $d_{0}$ is a reference distance corresponding to a point located in the far field of the transmit antenna, $\lambda_{p}$ is the wavelength of the propagating signal, $\rho_{c}$ is the path loss exponent and $X_{\sigma_{c}}∼N\left(0,\sigma_{c}^{2}\right)$ . The power loss (in dB) is therefore a Gaussian random variable with mean ${\text{μ}}_{c}$ and variance $\sigma_{c}^{2}$ , *i.e.*, $P_{L}∼N\left(\mu_{c},\sigma_{c}^{2}\right)$ .

The wireless channel represents an unreliable bit pipe for the data link layer, with instantaneous Shannon capacity $C=W\log_{2}\left(1+P_{r}/N_{0}W\right)\text{bps}$ , where *W* is the channel bandwidth and $P_{r}$ is the signal power received by the fusion center. Using Shannon’s coding theorem and given the state-of-the-art coding schemes that approach the Shannon capacity, we can approximately assume that the fusion center can perform error-free decoding for any transmission with bit rate R<C , *i.e.*, the channel is considered “ON” when R<C and “OFF” otherwise, giving rise to the two-state channel model akin to [9]. This condition is equivalent to $P_{r}\begin{array}{c} \text{ON}\\ ≷\\ \text{OFF} \end{array}N_{0}W\left(2^{\frac{R}{W}}-1\right)$ . Using Equation (2), noting that $P_{r}=P_{t}10^{-P_{L}/10}$ , where $P_{t}$ is the average signal power transmitted by the local sensor, and using the result that $P_{L}∼N\left(\mu_{c},\sigma_{c}^{2}\right)$ , we get the probability of the channel being “ON” during sensor *i* transmission:

(3) $$\begin{array}{c} P\left[\text{channel}\;\text{is}\;\text{ON}\right]=\lambda_{c}=\Phi \left[\frac{1}{\sigma_{c}}\left(10\log\frac{P_{t}}{N_{0}W\left(2^{\frac{R}{W}}-1\right)}-\mu_{c}\right)\right] \end{array}$$

where Φ(.) is the cumulative distribution function for the standard normal PDF. We note that the CSI relevant to our model is represented by the statistics $\sigma_{c},\mu_{c}$ and $N_{0}$ . These statistics are required to be estimated by each sensor, and no instantaneous channel state information is required for the XCP design. Since we assume fixed nodes and a slowly-varying channel, the estimation process could be executed less frequently to save sensor node resources. This is particularly important in wireless sensor networks, since the estimation of the channel state is both time and power consuming.

It should be highlighted that the large-scale fading model presented here allows us to obtain the closed form solution in Equation (3). More complex fading models, e.g., small-scale fading, can be integrated similarly, but they may allow only numerical solutions.

Equation (3) represents our model for the wireless channel in terms of our first system design variable; the communication rate for each sensor *R*. In the following subsections, where other layers are modeled, the design variable *R* will be coupled to other system parameters and design variables.

### 4.2. Media Access Control Protocol Model

In this section, we extend the probabilistic channel model to include the collision phenomenon of the MAC protocol. We assume a slotted ALOHA multi-access communication protocol between each parent node and its child nodes, where each packet requires one time slot for the transmission, all time slots have the same length, and all transmitters are synchronized. We consider a simplified version of the MAC protocol, where there is no retransmission of data in case of a collision or physical channel drops, since outdated information is not useful for real-time detection and estimation applications. In the rest of the paper, we refer to the simplified version of slotted ALOHA with no retransmission as S-ALOHA-NR. Furthermore, we assume that the sub-trees composed of each parent and its immediate child nodes do not interfere with each other. This could be achieved in practice by using different wireless channels for transmission, or it may be as a result of the physical separation between sub-trees, such that sub-tree transmissions get attenuated before interfering with other transmissions.

**Figure 5:**
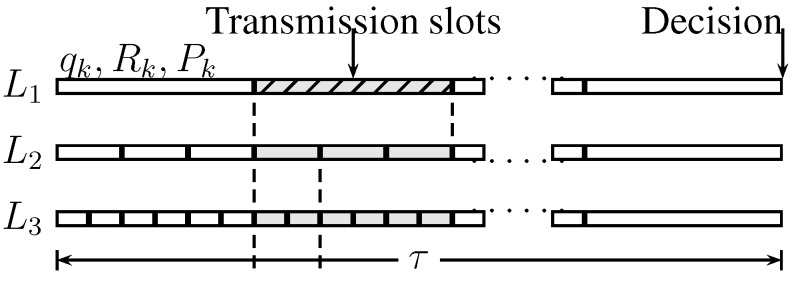
Detection cycle for the tree network.

The detection cycle, demonstrated in Figure 5, has length *τ*, which defines the delay for detection. The detection cycle is divided into a number of transmission slots $L_{i}$ , for nodes at the same depth *i* of the tree, and sharing a common parent. The relationship between the number of slots for consecutive depths is given by $L_{i+1}=m_{i}L_{i}$ , where $m_{i}$ is a positive integer. In the following discussion, we designate the set of all child nodes for sensor *k* by $C_{k}$ and the set of all siblings (excluding sensor *k*) by $B_{k}$ .

#### 4.2.1. Direct Transmission

At the beginning of every time slot, each local sensor *k* collects a number of observations $n_{k}$ and forms an information packet for transmission over the wireless channel. The sensor then attempts to transmit to its parent with probability $q_{k}$ , transmission power $P_{k}$ and communication rate $R_{k}$ . The sensor attempts transmission at each time slot, despite the status of its previous transmission attempts. The final decision is taken at the fusion center using the information received during the detection cycle. The process repeats for every detection request initiated by the fusion center.

**Figure 6:**
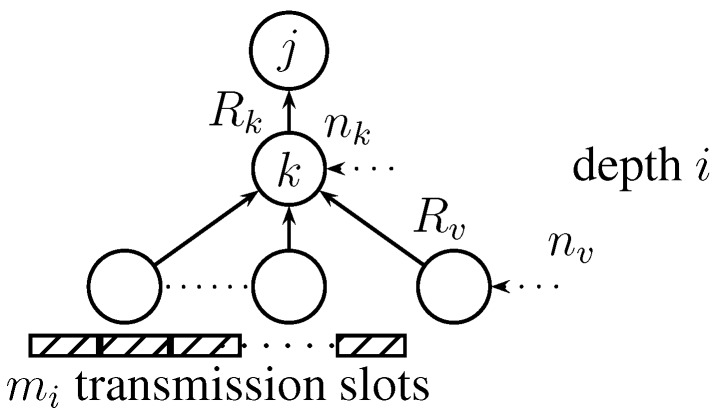
Communication rate calculation for node *k* at tree depth *i*.

The communication rate for sensor *k* at tree depth *i* could be expressed with the aid of Figure 6 as follows:

(4) $$\begin{array}{c} R_{k}=\frac{bL_{i}n_{k}}{\tau }+\frac{1}{m_{i}}\sum_{v\in C_{k}}Z_{v}R_{v},\;\sum_{v\in C_{k}}Z_{v}=m_{i} \end{array}$$

where *b* is the number of encoding bits for each observation, which is fixed, and $Z_{v}$ is the number of times the child sensor *v* successfully transmitted during the $m_{i}$ time slots associated with one time slot at level *i*. The first term in Equation (4) represents the information collected by the sensor node and vanishes if the node functions as a relay node for its child nodes. The second term represents the information received from the child nodes and vanishes for leaf nodes. We note here that the design variable is $n_{k}$ , the number of observations collected at each time slot by sensor *k*.

Now, we calculate the overall probability of a successful packet transmission, including the wireless channel effect. We note from Equation (4) that the communication rate of intermediate nodes is a random variable, being dependent on the information received from its child nodes. Accordingly, accurate formulation for the problem requires modification of Equation (3) to include the randomness of the communication rate. Unfortunately, no closed form solution could be obtained for the channel ON probability in this case. Even if we approximated the sum in the second term of Equation (4) by a Gaussian random variable using the central limit theorem and used the approximation of *R* to derive the new probability of success, we would obtain, after some manipulations, a sum of two log normal random variables, which does not have a closed-form probability distribution. Therefore, to keep the analysis tractable, we resort to a suboptimal solution, where the communication rate for each node is represented by its expected value. Accordingly, Equation (3) is still applicable, where *R* represents the average communication rate. Now, at any given time slot, the probability of a single packet transmission by sensor *k* is given by $q_{k}\prod_{v\in B_{k}}\left(1-q_{v}\right)$ . Further, this packet will be successfully received by the parent node if the state of the physical channel between the child node *k* and the parent node is “ON” during this time slot. Therefore, using Equation (3), the total probability of a successful packet transmission by sensor *k* is given by:

(5) $$\lambda_{k}=q_{k}\left[\prod_{v\in B_{k}}\left(1-q_{v}\right)\right]\Phi \left[\frac{10}{\sigma_{c}^{k}}\log\frac{P_{t}^{k}}{N_{0}W\left(2^{\frac{{\bar{R}}_{k}}{W}}-1\right)}-\frac{\mu_{c}^{k}}{\sigma_{c}^{k}}\right]$$

where ${\bar{R}}_{k}$ is obtained by taking the expected value for Equation (4), noting that $Z_{v}$ is a binomially-distributed random variable with $E\left[Z_{v}\right]=m_{i}\lambda_{v}$ and assuming that the random variables *Z* for channels at different levels of the tree network are independent:

(6) $$\begin{array}{c} {\bar{R}}_{k}=\frac{bL_{i}n_{k}}{\tau }+\sum_{v\in C_{k}}\lambda_{v}{\bar{R}}_{v} \end{array}$$

Equation (5) represents our extended model for the communication channel, where the second design variable, the retransmission probability for each sensor, *q*, is introduced.

#### 4.2.2. In-Network Processing

After each sensor *k* collects its $n_{k}$ observations in slot *i*, it calculates its LLR:

(7) $$\begin{array}{c} z_{k}=\frac{\mu^{k}}{\sigma_{s}^{k^{2}}}\sum_{j=1}^{n_{k}}x\left[j,i\right] \end{array}$$

where *x* is a Gaussian random variable (as explained in Section 4.4). There is no loss of optimality in this process, since the LLR is optimal at the fusion center as observations are independent across sensors [17]. The LLR is then quantized using $b_{k}$ bits, to obtain the discrete random variable $y_{k}$ :

(8) $$y_{k}=Q\left(z_{k};b_{k}\right)$$

This quantized version is transmitted to the parent node. Each sensor node forwards the quantized LLR of its descendants without further quantization, in addition to its own quantized LLR, to the next parent node. The process repeats until all observations arrive at the fusion center. Similar to Equation (6), the communication rate for each sensor is given by:

(9) $${\bar{R}}_{k}=\frac{b_{k}L_{i}}{\tau }+\sum_{v\in C_{k}}\lambda_{v}{\bar{R}}_{v}$$

We note here that the number of quantization bits is the design variable, and it may vary for each sensor. The decision on how many quantization bits will be used is dependent on the sensor quality measures. A large number of quantization bits reduces the loss in the signal to noise ratio, but increases the probability of packet loss.

### 4.3. Energy Model

In this section, we develop an energy model that enables us to replace the transmission power, $P_{t}$ , in Equation (5) by the sensor energy and other network parameters. To formulate the energy model for each sensor, we first introduce the definition for the network lifetime. The network lifetime L could be defined as the average time span from the deployment to the instant when the network can no longer perform the task [13]. The network lifetime could be expressed as:

(10) $$\begin{array}{c} L=\frac{E^{0}-E^{w}}{f_{r}E^{r}} \end{array}$$

where $E^{0}=\sum_{i=1}^{N}e_{i}^{0}$ is the total initial energy in all sensors at the time of deployment, $E_{w}=\sum_{i=1}^{N}e_{i}^{w}$ is the total energy remaining in sensor nodes when the network cannot perform the assigned task, $f_{r}$ is the average sensor reporting rate defined here as the number of detection cycles per unit time and $E^{r}=\sum_{i=1}^{N}e_{i}^{r}$ is the expected energy consumed by all sensors in one detection cycle. The total energy remaining is defined for our detection problem as the energy required to achieve a minimum pre-specified value for the detection performance measure.

In this work, we resort to a simple energy formulation. First, we assume that $e_{i}^{w}$ is the energy remaining in the sensor battery when the sensor is not capable of operating its electronic circuits for computations and communication, which is fixed and known for each sensor. Second, we assume that the reporting energy for each sensor $e_{i}^{r}$ is a fixed percentage of its net useful energy at the time of sensor deployment. Using these two assumptions, we get the following expression for the energy consumed by each sensor *k* in one detection cycle:

(11) $$\begin{array}{c} e_{k}^{r}=\frac{e_{k}^{0}-e_{k}^{w}}{f_{r}L} \end{array}$$

which could be calculated for any desired network lifetime L . The total energy consumed by each sensor is divided between transmission and reception (except for leaf nodes). By assuming that the energy consumed in the reception process is proportional to the detection cycle time with proportionality constant *α* and by noting that the expected number of transmissions by sensor *k* during a detection cycle is $L_{i}q_{k}$ , we get:

(12) $$\begin{array}{cc} P_{t}^{k} & =\frac{(e_{k}^{r}/\tau )-\alpha }{q_{k}}=\frac{1}{q_{k}}\left(p_{k}-\alpha \right) \end{array}$$

where $p_{k}$ is the average transmission power over one detection cycle, which summarizes the residual energy information (REI) for each sensor. Using Equation (12) in Equation (5), we get:

(13) $$\begin{array}{c} \lambda_{k}=q_{k}\left[\prod_{v\in B_{k}}\left(1-q_{v}\right)\right]\Phi \left[a_{k}-\left(\frac{10}{\sigma_{c}^{k}}\right)\log q_{k}\left(2^{\frac{{\bar{R}}_{k}}{W}}-1\right)\right] \end{array}$$

where $a_{k}=\frac{1}{\sigma_{c}^{k}}\left(10\log\frac{p_{k}-\alpha }{N_{0}W}-\mu_{c}^{k}\right)$ . We note that $\alpha <p_{k}=e_{k}^{r}/\tau$ for the sensor to be able to transmit the information. In addition, α=0 for leaf nodes.

This energy formulation simplifies the analysis, as the reporting energy $e^{r}$ for each sensor is preallocated. In general, however, we can include the energy allocation problem in our formulation, *i.e.*, finding optimal $e^{r}$ values for all sensors that maximize the detection performance while guaranteeing a minimum network lifetime.

### 4.4. Sensing Model

In the previous sections, the physical and network layer models are developed. Intuitively, this model captures the probability that the transmitted sensor information will be received successfully at the parent node. Now, we turn our attention to develop the model for the sensor information itself. In Section 4.5, the detection performance measure will bring the two models together, defining our target optimization problem. We focus our work on detection using radar-like sensors, where ultra-wideband technology is employed for target detection [18]. These sensors use micro-power impulses rather than continuous narrow-band transmissions used in conventional radars. After front-end signal processing, signal amplitude is used for detection. Therefore, the observation at sensor *k*, located at $d_{k}$ distance from the target located in a specific resolution cell, could be expressed as:

(14) $$\begin{array}{c} x_{k}=\zeta \frac{\epsilon_{tr}}{{\left(2d_{k}\right)}^{\eta /2}}+w_{k} \end{array}$$

where *ζ* is a known reflection coefficient at the target, $\epsilon_{tr}$ is the amplitude of the signal transmitted by the active sensor (illuminating signal), $2d_{i}$ is the round trip distance traveled by the signal, *η* is a known attenuation coefficient, typically between two and four, and $w_{k}$ is an additive white Gaussian noise with zero mean and variance ${\left(\sigma_{s}^{k}\right)}^{2}$ . This information is shown in Figure 1 for one sensor.

The detection problem could be defined as the following binary hypothesis testing problem, for each time slot *i*:

$$\begin{array}{cc} H_{0}:x_{k}\left[j,i\right]=w_{k}\left[j,i\right]\; & j=1,2,\ldots ,n_{k} \end{array}$$

(15) $$\begin{array}{c} H_{1}:x_{k}\left[j,i\right]=\mu^{k}+w_{k}\left[j,i\right]\;j=1,2,\ldots ,n_{k} \end{array}$$

where $\mu^{k}=\epsilon /d_{k}^{\eta /2}$ and $n_{k}$ is the number of observations obtained by sensor *k* at each time slot. We note that noise samples are independent across sensors, *i.e.*, the observations at local sensors are independent across time and space, but not necessarily identically distributed, since some sensors may be closer to the measured phenomenon, and noise variances are assumed unequal.

Based on the given sensing model, we next derive the detection performance measure for the two transmission schemes, which represents the objective function to be optimized.

### 4.5. Measurement of Detection Performance

Intuitively, the detection performance measure should depend both on the quality of information, as well as the quality of the communication channel. In this section, we present a measure for the detection performance and express it in terms of the other system parameters, using the models developed in the previous sections. One of the widely-used performance measures for detection applications is the receiver operating characteristics (ROC) curve [19]. The curve relates the probability of detection $P_{D}$ to the probability of false alarm $P_{FA}$ for different threshold values *γ* of the detector. For example, for the centralized shift-in-mean Gaussian detection problem, where all observations are available at the fusion center, the ROC curve is expressed as:

(16) $$\begin{array}{c} P_{D}=Q\left(Q^{-1}\left(P_{FA}\right)-\left(\frac{\mu_{1}-\mu_{0}}{\sigma }\right)\right) \end{array}$$

where Q[.]=1−Φ[.] is the complementary cumulative distribution function for the standard normal PDF, $\mu_{1}-\mu_{0}$ is the shift-in-mean value and $\sigma^{2}$ is the measurement variance. For our detection problem, the ROC curve cannot be expressed by one equation as in Equation (16), due to the complexity of the equations. Furthermore, optimization with respect to the ROC is computationally prohibitive. Therefore, we adopt the deflection coefficient, a closely-related performance measure that leads to a computationally less-intensive problem, defined as [19]:

(17) $$\begin{array}{c} D^{2}=\frac{{\left(E\left[V;H_{1}\right]-E\left[V;H_{0}\right]\right)}^{2}}{\text{var}[V;H_{0}]} \end{array}$$

The deflection coefficient is a measure of the separation between the two probability density functions under the two hypotheses. Under Gaussian assumptions, it is known that maximizing the deflection coefficient leads to maximization of the detection performance in terms of the ROC curve [20]. In fact, it can be shown that for the centralized shift-in-mean Gaussian detection problem, the ROC curve in Equation (16) could be expressed as [19]:

(18) $$\begin{array}{c} P_{D}=Q\left(Q^{-1}\left(P_{FA}\right)-\sqrt{D^{2}}\right) \end{array}$$

Under non-Gaussian assumptions, there is no general result that enhancement of the deflection coefficient will lead to a better performance in terms of the ROC curve. However, it is likely that more separation between the two density functions will lead to a better detection performance.

#### 4.5.1. Direct Transmission

The following proposition defines the optimal test statistic at the fusion center for the tree sensor network. The detailed proof is listed in Appendix A.

**Proposition 1.**  *The optimal test statistic at the fusion center for the slotted ALOHA tree network with depth l and direct transmission scheme is given by the recursive formula:*

(19) $$\begin{array}{ll} V_{k} & =\sum_{i_{k}=1}^{m_{k}}\sum_{v\in C_{k}}\sum_{j=1}^{n_{v}}r_{v}\left[i_{FC}\ldots i_{k}\right]\left[\left(\frac{\mu^{v}}{\sigma_{s}^{v^{2}}}\right)x_{v}\left[j,i_{FC}\ldots i_{k}\right]+V_{v}\right]\;,k=1,2,\ldots \mathrm{FC}\\ V_{v} & =0\;\mathrm{if}\;k\;\mathrm{is}\;a\;\mathrm{leaf}\;\mathrm{node} \end{array}$$

*where* $m_{k}$ *is the number of communication slots at the tree depth corresponding to sensor k*, $r_{v}\left[i_{FC}\ldots i_{k}\right]$ *is a Bernoulli random variable representing the success (* $r_{v}=1$ *) or failure (* $r_{v}=0$ *) of receiving a packet from sensor v in the communication slot sequence* $i_{FC}\ldots i_{k}$ . *The sample space and probability measure of* $r_{v}$ *are defined as* $\Omega_{r_{v}}=\left\{0,1\right\}$ *and* $P\left[r_{v}=1\right]=\lambda_{v}$ , *respectively, where* $\lambda_{v}$ *is given by Equation (5). Complete nomenclature for the system model is shown in Table 1*.

The expression in Equation (19) is simply a weighted sum of the observations received at the fusion center. The complexity of the equation comes from the fact that successful reception of the observations of child nodes at the fusion center depends on the success of the transmission of all parent nodes up to the fusion center.

**Table 1:** Nomenclature for the ALOHA tree sensor network.

Param.	Description
$\mu_{c}^{i}$	Mean path loss for sensor *i*
$\sigma_{c}^{i}$	Path loss standard deviation for sensor *i*
*W*	Communication channel bandwidth
$P_{t}^{i}$	Transmission power for sensor *i*
$p_{i}$	Average transmission power for sensor *i* over one detection cycle
$N_{0}$	Noise power spectral density
${\bar{R}}_{i}$	Average communication rate for sensor *i*
*b*	Number of encoding bits/observation
$L_{i}$	Number of transmission slots at tree depth *i*
$m_{i}$	Number of sub-slots at tree depth i+1 for each slot at tree depth *i*
$n_{i}$	Number of observations sampled by sensor *i*
*l*	Tree depth
*τ*	Delay for detection
$\lambda_{i}$	Successful packet transmission probability for sensor *i*
$q_{i}$	Retransmission probability for sensor *i*
L	Sensor network lifetime
$L_{i}$	Sensor *i* lifetime
$e_{i}^{0}$	Initial energy in sensor *i* battery
$e_{i}^{w}$	Wasted energy remaining in sensor *i* battery
$e_{i}^{r}$	Reporting energy for sensor *i*
$f_{r}$	Reporting frequency for the sensor network
$\alpha_{e}$	Percentage of net useful energy used in reporting
α	Proportionality constant for receiving energy
*ϵ*	Amplitude of emitted signal at the detected object
$d_{i}$	Distance between sensor *i* and the object
*η*	Attenuation coefficient for object signal
$x_{i}\left[j,k\right]$	Observation number *j* at time slot *k* for sensor *i*
$c^{i}={\left(\mu^{i}/\sigma_{s}^{i}\right)}^{2}$	Detected object signal to noise ratio at sensor *i*
*V*	Test statistic at the fusion center
*N*	Total number of wireless sensors
$r_{i}\left[k\right]$	Success or failure of sensor *i* transmission in slot *k*
$D^{2}$	Deflection coefficient
$C_{k}$	Set of all child nodes for sensor *k*
$B_{k}$	Set of all sibling nodes for sensor *k*

**Proposition 2.**  *The deflection coefficient for the detector in Equation (19) is given by the recursive formula:*

(20) $$\begin{array}{l} D^{2}=L_{1}G_{FC}\\ G_{k}=\sum_{v\in C_{k}}\lambda_{v}\left[n_{v}c_{v}+m_{v}G_{v}\right]\;,k=1,2,\ldots \mathrm{FC}\\ G_{v}=0\;\mathrm{if}\;k\;\mathrm{is}\;a\;\mathrm{leaf}\;\mathrm{node} \end{array}$$

*where* $c_{v}={\left(\mu^{v}/\sigma_{s}^{v}\right)}^{2}$ .

The proof is found in Appendix B. We note that the quantity $n_{v}c_{v}$ is a measure of the QoI for each sensor. Using Equation (6) in Equation (20), we obtain our objective function:

(21) $$\begin{array}{l} D^{2}=\frac{\tau }{b}J_{FC}\\ J_{k}=\sum_{v\in C_{k}}\lambda_{v}\left[R_{v}\left(c_{v}-c_{k}\right)+J_{v}\right]\;,k=1,2,\ldots \text{FC}\\ J_{v}=0\;\mathrm{if}\;k\;\mathrm{is}\;a\;\mathrm{leaf}\;\mathrm{node},c_{FC}=0 \end{array}$$

#### 4.5.2. In-Network Processing

To obtain the optimal test statistic, we need to take the LLR for the discrete random variables $Y_{i}$ in Equation (8) at the fusion center. Unfortunately, this problem does not have a closed form solution, and the detector performance is usually approximated using different statistical techniques [21]. We resort to the following suboptimal statistic, as it is similar to the one in Equation (19) for the direct observation system, which facilitates the performance comparison:

(22) $$\begin{array}{ll} V_{k} & =\sum_{i_{k}=1}^{m_{k}}\sum_{v\in C_{k}}r_{v}\left[i_{FC}\ldots i_{k}\right]\left[y_{v}\left[i_{FC}\ldots i_{k}\right]+V_{v}\right]\;,k=1,2,\ldots \text{FC}\\ V_{v} & =0\;\mathrm{if}\;k\;\mathrm{is}\;a\;\mathrm{leaf}\;\mathrm{node} \end{array}$$

Now, to find the deflection coefficient for the statistic in Equation (22), we need to calculate the expectation of *V* under both $H_{0}$ and $H_{1}$ , in addition to its variance under $H_{0}$ . We first need to define the quantization function in Equation (8). We adopt the following quantizer:

(23) $$\begin{array}{c} Q\left(z\right)=\Delta \left(\left\lfloor \frac{z}{\Delta }\right\rfloor +\frac{1}{2}\right) \end{array}$$

where Δ is the quantizer step size. We have the following proposition.

**Proposition 3.**  *The deflection coefficient of the test statistic in Equation (22), with the quantizer in Equation (23), is given by:*

(24) $$\begin{array}{ll} D^{2} & =L_{1}\frac{G_{FC}^{2}}{G_{FC}^{\prime }}\\ G_{k} & =\sum_{v\in C_{k}}\lambda_{v}\left[n_{v}c_{v}+\delta_{v}+m_{v}G_{v}\right]\;,k=1,2,\ldots FC\\ G_{k}^{\prime } & =\sum_{v\in C_{k}}\lambda_{v}\left[n_{v}c_{v}+\delta_{v}^{\prime }+m_{v}G_{v}\right]\\ G_{v} & =G_{v}^{\prime }=0\;\mathrm{if k is a leaf node} \end{array}$$

*where:*

(25)δ=Δπ∑k=1∞1ksin2πknΔμσs2e−2πkΔ2nμσs2δ′=Δπ2∑k1=1∞∑k2=1∞12k1k2cos2π(k1−k2)nΔμσs2e−2π(k1−k2)Δ2nμσs2−(26) cos2π(k1+k2)nΔμσs2e−2π(k1+k2)Δ2nμσs2

The proof is listed in Appendix C. The expression for the deflection coefficient in Equation (24) is not amenable to optimization. However, we note that both the mean and variance degrade exponentially with the quantizer step size Δ. Since Δ is inversely proportional to the number of quantization bits, $b_{k}$ , we can approximate the degradation in the signal to noise ratio for each sensor *k* by:

(27) $$\begin{array}{c} S=n_{k}{\left(\frac{\mu_{k}}{{\sigma^{k}}^{2}}\right)}^{2}\left(1-2^{-\beta b_{k}}\right) \end{array}$$

where β specifies the decay rate and depends on the range of the quantizer, as well as the quantizer design. Now, we use the degraded SNR in Equation (27) to define our approximate deflection coefficient as:

(28) $$\begin{array}{ll} D^{2} & =L_{1}G_{FC}\\ G_{k} & =\sum_{v\in C_{k}}\lambda_{v}\left[n_{v}c_{v}^{\prime }+m_{v}G_{v}\right]\;,k=1,2,\ldots \text{FC}\\ G_{v} & =0\;\mathrm{if}\;k\;\mathrm{is}\;a\;\mathrm{leaf}\;\mathrm{node} \end{array}$$

where $c_{v}^{\prime }={\left(\mu^{v}/\sigma_{s}^{v}\right)}^{2}\left(1-2^{-\beta b_{v}}\right)$ .

For comparison purposes with the direct transmission approach, we use the same number of observations for each sensor and the same number of slots as in the direct transmission case. From Equation (6), we obtain:

(29) $$\begin{array}{c} L_{i}n_{v_{i}}=\frac{\tau }{b}\left({\bar{R}}_{v_{i}}-\sum_{v_{j}\in C_{v_{i}}}\lambda_{v_{j}}{\bar{R}}_{v_{j}}\right)=\frac{\tau }{b}\left({\bar{R}}_{v_{i}}-r_{v_{i}}\right)=\frac{\tau }{b}u_{v_{i}} \end{array}$$

where ${\bar{R}}_{v_{i}}$ is the average communication rate for sensor $v_{i}$ , $r_{v_{i}}$ is the average communication rate for $C_{v_{i}}$ and *b* is the number of quantization bits in the direct transmission case. The values of these three quantities are obtained from the solution of the optimization problem in the direct transmission case. The objective function could then be expressed as:

(30) $$\begin{array}{ll} D^{2} & =\frac{\tau }{b}J_{FC}\\ J_{k} & =\sum_{v\in C_{k}}\lambda_{v}\left[u_{v}c_{v}^{\prime }+J_{v}\right]\;,k=1,2,\ldots \text{FC}\\ J_{v} & =0\;\mathrm{if}\;k\;\mathrm{is}\;a\;\mathrm{leaf}\;\mathrm{node} \end{array}$$

## 5. System Design for Optimal Detection

In Section 4, we developed an integrated model for the detection system, obtained an expression for the detection performance measure (the deflection coefficient), and defined the design constraints. Now, we need to solve the optimization problem to obtain the system design variables (retransmission probability *q* and communication rate *R*) for each sensor for both direct transmission and in-network processing designs.

### 5.1. Direct Transmission

From Equation (21), the optimization problem could be expressed as follows:

maxq,R¯τb∑v1∈Cfλv1R¯v1cv1+∑v2∈Cv1λv2R¯v2(cv2−cv1)+…+∑vl∈Cvl−1λvlR¯vl(cvl−cvl−1)…(31)  s.t.0≤qi≤1,R¯i≥∑v∈CiλvR¯vi=1:N

where:

(32) $$\begin{array}{ll} \lambda_{v} & =q_{v}\left[\prod_{k\in B_{v}}\left(1-q_{k}\right)\right]\Phi \left[a_{v}-\left(\frac{10}{\sigma_{c}^{v}}\right)\log q_{v}\left(2^{\frac{{\bar{R}}_{v}}{W}}-1\right)\right] \end{array}$$

(33) $$\begin{array}{ll} a_{v} & =\frac{1}{\sigma_{c}^{v}}\left(10\log\frac{p_{v}-\alpha }{N_{0}W}-\mu_{c}^{v}\right),c_{v}={\left(\frac{\mu^{v}}{\sigma_{s}^{v}}\right)}^{2} \end{array}$$

**Figure 7:**
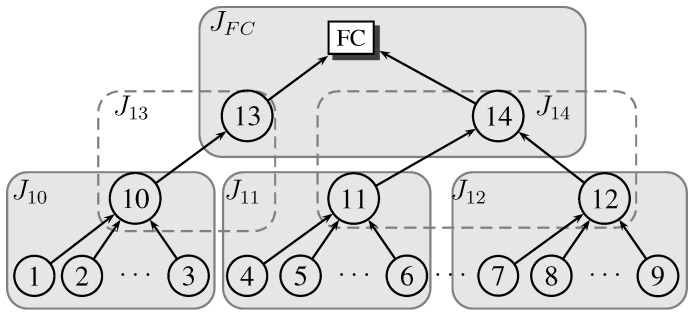
Hierarchical optimization for the transmission control policy (XCP) design problem.

The last constraint guarantees that intermediate nodes can at least relay the observations of their child nodes. This constraint reduces to ${\bar{R}}_{i}\geq 0$ for leaf nodes. Although this problem could be solved by existing algorithms (e.g., interior point methods) for a local maximum, we note that the objective function in Equation (31) gets more complicated as the tree depth increases. Adding the fact that all design variables have to be propagated back to tree nodes, a more practical approach is clearly needed. If we look at the objective function expression in Equation (21), we note that it reflects the tree hierarchy, *i.e.*, the last term in the expression represents the contribution of the leaf nodes, preceded by the contribution of the parents of the leaf nodes, and so on, until reaching the sensor nodes at the top level of the tree (depth = 1). This structure of the objective function suggests a local optimization approach for the problem, where we start by optimizing $J_{v}$ for sensors at depth l−1 and continue the local optimization recursively using Equation (21), until reaching the fusion center. This approach is practical since the solution of each local optimization problem could be carried out locally at each parent node. The solution approach is illustrated in Figure 7.

By substituting Equation (13) in Equation (21), we can express the local optimization problem at parent node *k* as follows:

maxq,R¯∑v∈Ckqv∏i∈Bv(1−qi)R¯v(cv−ck)+JvΦav−10σcvlogqv2R¯vW−1(34)   s.t.0≤qv≤1,R¯v≥∑u∈CvλuR¯u=rv

**Figure 8:**
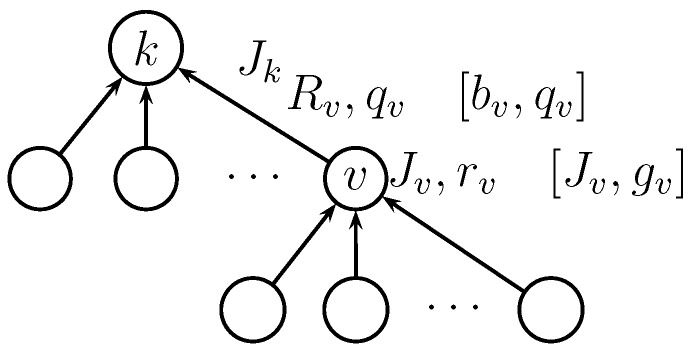
Notation for the local optimization problem. In-network processing parameters are shown between brackets.

We note that $J_{v}$ and $r_{v}$ are fixed values, obtained from solving the local optimization problems at lower levels in the hierarchy. The notation for the local optimization problem is illustrated in Figure 8.

Let the number of child nodes for sensor *k* be $N_{k}$ , and denote the decision variables by:

(35) $$\begin{array}{c} x=\left[\begin{array}{cccccccc} q_{1} & q_{2} & \ldots & q_{N_{k}} & {\bar{R}}_{1} & {\bar{R}}_{2} & \ldots & {\bar{R}}_{N_{k}} \end{array}\right] \end{array}$$

where $x\in R^{2N_{k}}$ , and the objective function by J(x) , then the optimization problem could be rewritten as:

(36) $$\begin{array}{c} \min_{x}-J\left(x\right)\;\text{subject}\;\text{to}\;Ax\geq b \end{array}$$

where:

(37) $$\begin{array}{c} A={\left[\begin{array}{ccc} I & -I & 0\\ 0 & 0 & I \end{array}\right]}^{T},\;b={\left[\begin{array}{ccc} 0 & -1 & r \end{array}\right]}^{T} \end{array}$$

*I* is the identity matrix, 0(1) is the vector/matrix of all zeros (ones) with appropriate dimensions and $r={\left[\begin{array}{cccc} r_{1} & r_{2} & \ldots & r_{N_{k}} \end{array}\right]}^{T}$ . Although the objective function is not convex, we note that the inequality constraints are linear. Therefore, the Karush–Kuhn–Tucker (KKT) conditions are necessary conditions for a local maximizer of the objective function [22]. We first form the Lagrangian:

(38) $$\begin{array}{c} L\left(x,\nu \right)=-J\left(x\right)-\nu^{T}\left(Ax-b\right) \end{array}$$

where *ν* is the vector of Lagrange multipliers, defined as:

(39) $$\begin{array}{cc} \nu^{T}=[ & \begin{array}{ccccccccc} \nu_{q_{1}^{0}} & \ldots & \nu_{q_{N_{k}}^{0}} & \nu_{q_{1}^{1}} & \ldots & \nu_{q_{N_{k}}^{1}} & \nu_{{\bar{R}}_{1}} & \ldots & \nu_{{\bar{R}}_{N_{k}}} \end{array}] \end{array}$$

$\nu_{q_{i}^{0}}$ and $\nu_{q_{i}^{1}}$ are the Lagrange multipliers for the retransmission probability constraint, and $\nu_{{\bar{R}}_{i}}$ is the Lagrange multiplier for the communication rate constraint in Equation (34). We denote the primal and dual optimal points by $x^{*}$ and $\nu^{*}$ , respectively. The KKT conditions are thus given by:

(40)−∇J(x*)−ATν*=0(Stationarity)(41)ν*T(Ax*−b)=0(Comp.slackness)(42)(Ax*−b)⪰0(Primalfeasibility)(43)ν*⪰0(Dualfeasibility)(44)−ZT∇2J(x*)Z⪰0

where *Z* is a null-space matrix for the matrix of active constraints at $x^{*}$ and ⪰ represents componentwise inequality for vectors and positive-semidefiniteness for matrices. We present the following proposition without proof, since the result could be obtained by straightforward manipulation of the KKT conditions.

**Proposition 4.**  *The maximum value of the objective function in Equation (34) occurs either when one sensor transmits with probability one and all of other sensors remain silent or at a stationary point of the objective function, i.e., at* $x^{*}$ *where* $\nabla J(x^{*})=0$ .

Since we may have multiple stationary points in the interior of the objective function domain, the proposition does not guarantee obtaining the global maximum. However, the proposition is still useful for the following reasons: (1) it avoids the case where the optimization algorithm may terminate at the local maximum $q_{i}=1,q_{j}=0$ , while a better local maximum may be at one of the stationary points and; (2) it provides information about the choice of the initial point for the optimization algorithm, where initial points near the corner points $q_{i}=1,q_{j}=0$ have to be avoided.

The solution of the optimization problem for the tree network with direct transmission scheme is summarized in Algorithm 1, where Proposition 4 is used, and the called optimization algorithm is any appropriate optimization method, e.g., the interior point method. The algorithm for the tree network with in-network processing could be developed similarly.
**Algorithm 1** Optimization problem solution for the tree network. Create tree object *T* with system parameter values **for** (*k* = *T* .BreadthTrav)! = null **do** {traverses T, returns a pointer to current node}  **if**
*k* is a Leaf node **then**   *J_k_* = 0, *r_k_* = 0   continue  **end if**  **for** (*V*=*T_k_* .ChildTrav)!= null **do** {traverses child nodes, breadth-first, returns a pointer to current node}   **C** = [**C**
*C_v_* ], J = [**J**
*J_v_* ], **r** = [**r**
*r_v_* ]  **end for**  OptFunc(*k,*
**J**, **C**, **r**, **q**, **R**, *J_k_, r_k_*) {call optimization algorithm for Equation (34)} **end for**

### 5.2. In-Network Processing

We note that the objective function in Equation (30) has the same recursive structure as the direct transmission design. Accordingly, we adopt the same local approach presented for the direct transmission scheme to solve for the optimal design variables. It can be shown that the local optimization problem at parent node *k* could be expressed as:

maxq,b∑v∈Ckqv∏i∈Bv(1−qi)Φav−10σcvlogqv2bv(L/τ)+gv/W−1uvμvσsv21−2−βbv+Jv(45)   s.t.0≤qv≤1,bv∈N

We note that $J_{v}$ and $g_{v}$ are fixed values, obtained from solving the local optimization problems at lower levels in the hierarchy. The notation for the local optimization problem with in-network processing is illustrated in Figure 8.

### 5.3. Handshaking Overhead

In order to run the optimization algorithm at each parent node to calculate the optimal communication parameters for child nodes, information has to be communicated from child nodes to each parent node. This communication overhead is ignored while developing the system model and the optimization algorithm. Ignoring the handshaking overhead is a valid assumption as long as the dynamics of the environment are slow, such that sensors’ information does not need to be communicated frequently to the relevant parent nodes. In this section, we derive an upper bound on the environment dynamics that enables us to ignore the handshaking overhead without affecting the model accuracy.

We measure the handshaking overhead by: (1) the total delay time taken by each parent sensor to collect the quality measures and optimization data from all child sensors in one handshaking cycle, $\tau_{h}$ and; (2) the total energy spent in the handshaking cycle, $e_{h}$ . We designate the communication rate and transmission power used by all child sensors during the handshaking process by $R_{h}$ and $P_{h}$ , respectively. We further designate the rate at which the environment changes by $f_{h}$ (handshaking cycles per day). Our objective is to derive an upper bound on $f_{h}$ for a given network. The delay condition could be expressed as:

(46) $$\begin{array}{c} \tau_{h}f_{h}<\alpha \left(\tau f_{r}\right) \end{array}$$

where α represents the allowed percentage of resources to be consumed in the handshaking process, such that the handshaking overhead could be ignored. To calculate $\tau_{h}$ , we assume IEEE 754 half-precision binary floating-point format (two bytes) to represent the quality measures and optimization data for each sensor [23]. According to Table 1 and the optimization algorithm in 1, we have seven values representing the sensor information to be communicated; CSI ( $N_{0},\mu_{c},\sigma_{c}$ ), QoI ( $\mu /\sigma_{s}$ ), REI, and optimization data J and r . Therefore, each sensor requires 14 bytes of payload. Assuming nine bytes of overhead, the total handshaking delay is given by:

(47) $$\begin{array}{c} \tau_{h}=\frac{23\times 8\times N_{c}}{R_{h}}=\frac{184N_{c}}{R_{h}} \end{array}$$

where $N_{c}$ is the number of child nodes for a specific parent node. Combining Equations (46) and (47):

(48) $$\begin{array}{c} f_{h}<\alpha \left(\frac{\tau f_{r}}{184N_{c}}\right)R_{h} \end{array}$$

The energy condition could be expressed as:

(49) $$\begin{array}{c} e_{h}f_{h}l<\alpha e^{0} \end{array}$$

where $e^{0}$ is the initial energy in each sensor battery, which is assumed the same for all sensors. The energy spent in the handshaking process by each sensor could be expressed as:

(50) $$\begin{array}{c} e_{h}=P_{h}\left(\frac{23\times 8}{R_{h}}\right)=184\left(\frac{P_{h}}{R_{h}}\right) \end{array}$$

Combining Equations (49) and (50):

(51) $$\begin{array}{c} f_{h}<\alpha \left(\frac{e^{0}}{184l}\right)\left(\frac{R_{h}}{P_{h}}\right) \end{array}$$

Equations (48) and (51) represent the two conditions that need to be satisfied to ensure that the derived model accuracy will not be affected by ignoring the handshaking overhead. These two conditions could be verified for any values of the design parameters $R_{h}$ and $P_{h}$ . However, since we ignored the channel drop probability during the handshaking process in the analysis, one more constraint is required to guarantee the minimum probability of successful transmission, *λ*, and, hence, reliable communication during the handshaking cycle. Since all sensors need to transmit during the handshaking process, we assume that TDMA is the protocol used during handshaking. Accordingly, we can use Equation (3) to get:

(52) $$\begin{array}{c} P_{h}=N_{0}W10^{0.1\left(\mu_{c}+\sigma_{c}\Phi^{-1}\left[\lambda \right]\right)}\left(2^{R_{h}/W}-1\right) \end{array}$$

and substituting in Equation (51):

(53) $$\begin{array}{c} f_{h}<\alpha \left(\frac{e^{0}10^{-0.1\left(\mu_{c}+\sigma_{c}\Phi^{-1}\left[\lambda \right]\right)}}{184N_{0}Wl}\right)\frac{R_{h}}{2^{R_{h}/W}-1} \end{array}$$

We note that $f_{h}$ needs to satisfy Equations (48) and (53) simultaneously. Since the right-hand side of Equation (48) is a monotonically-increasing function of $R_{h}$ and the right-hand side of Equation (53) is a monotonically-decreasing function of $R_{h}$ , the upper bound on $f_{h}$ is at the intersection of the two functions. Hence:

(54)Rh=Wlog21+e0Nc10−0.1μc+σcΦ−1[λ]N0Wlτfr(55)Ph=e0Nclτfr

and finally, the upper bound on $f_{h}$ is given by:

(56) $$\begin{array}{c} f_{h}<\alpha \left(\frac{\tau f_{r}W}{184N_{c}}\right)\log_{2}\left[1+\frac{e^{0}N_{c}10^{-0.1\left(\mu_{c}+\sigma_{c}\Phi^{-1}\left[\lambda \right]\right)}}{N_{0}Wl\tau f_{r}}\right] \end{array}$$

Accordingly, for any given sensor network, the analysis and the developed model could be used with sufficient accuracy as long as the environment dynamics do not require more than $f_{h}$ cycles/day, as calculated by Equation (56), to update each parent node. If the environment dynamics are much faster, then the handshaking overhead has to be included in the model development.

### 5.4. Optimization Problem Variants

The general optimization problem for decentralized detection with wireless sensor networks as presented here could be formulated as [24]:

(57) $$\begin{array}{c} \max_{P\subset P}Q\left(P;G,T,R,C\right) \end{array}$$

where *Q* represents the quality of information for the detection task, *P* is the network protocol parameter set, *G* is the detection strategy, *i.e.*, the signal processing algorithm for the observations, *T* is the network topology, *R* is the network reliability, and *C* is the network deployment cost, in terms of energy consumption and number of sensors. In this paper, P=[qR] represents the decision variables, $Q=D^{2}$ is the deflection coefficient, *G* is the detection strategy defined by the likelihood ratio test at the fusion center or the parent node, *T* is the tree network structure, *R* is the physical channel reliability, which is incorporated in the model, and *C* is the energy consumption as formulated in Section 4.3

Different variants of the general optimization problem could be expressed similarly. For example, if the lifetime is the main constraint in the problem, then the optimization problem could be expressed as:

(58) $$\begin{array}{c} \min_{P}C\left(P;G,T,R\right) \end{array}$$

To put the system model in this form for the given problem, the energy model in Section 4.3 has to be reformulated to express the network lifetime, L , as a function of network parameters. To do that, the relaxing assumption of pre-allocation of sensor energy has to be dropped.

The detection time is considered as a quality of information metric for the detection task. Therefore, the formulation in Equation (57) is applicable provided that *Q* represents the negative of the detection time (equivalently, minimization of the detection time). Expressing the system model for the given detection problem in terms of the detection time, *τ*, is straightforward. From Equation (31):

(59) $$\begin{array}{c} \tau =bD^{2}/\sum_{v_{1}\in C_{f}}\lambda_{v_{1}}\left[{\bar{R}}_{v_{1}}c_{v_{1}}+\sum_{v_{2}\in C_{v_{1}}}\lambda_{v_{2}}\left[{\bar{R}}_{v_{2}}\left(c_{v_{2}}-c_{v_{1}}\right)+\ldots +\sum_{v_{l}\in C_{v_{l-1}}}\lambda_{v_{l}}{\bar{R}}_{v_{l}}\left(c_{v_{l}}-c_{v_{l-1}}\right)\right]\ldots \right] \end{array}$$

For a given detection performance measure $D^{2}$ , minimization of *τ* is equivalent to maximization of the denominator in Equation (59); hence, this problem is equivalent to the one presented in the paper.

## 6. Performance Evaluation

**Figure 9:**
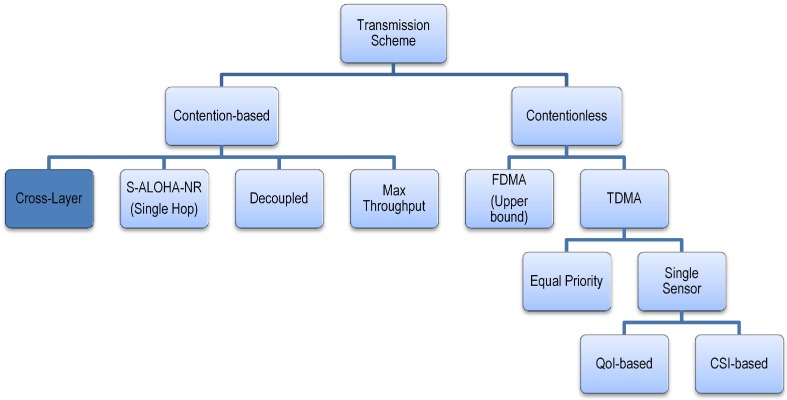
Transmission schemes for performance comparison.

**Figure 10:**
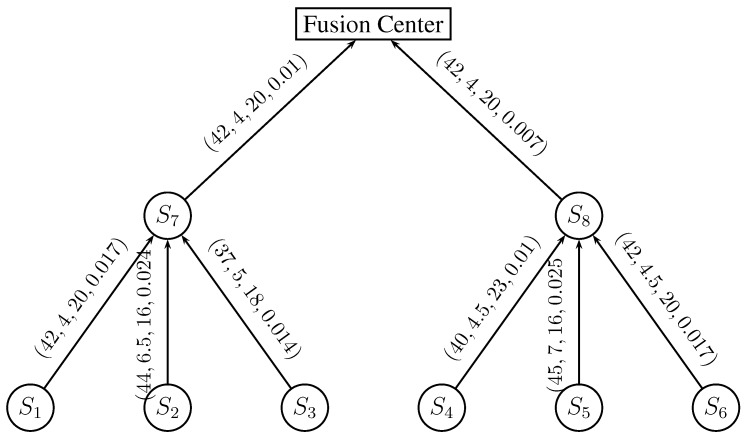
Tree detection network for the example problem. Labels on each edge represent $\mu_{c},\sigma_{c},e$ (in mJ), and the signal to noise ratio, respectively, for each source sensor.Tree detection network for the numerical example

We compare the cross-layer design approach to the design schemes presented in Figure 9. We use the tree network in Figure 10, with system parameters as indicated on the tree edges. We use $W=2\times 10^{3}$ Hz, $N_{0}=10^{-10}$ W/Hz, and b=16 bits. The comparison is performed using numerical optimization and Monte Carlo simulations (MCS) as follows:

Optimization: For each design scheme, the analytical expression for the deflection coefficient is used to form the optimization problem. The optimization problem is solved numerically for the presented example network and specific values for the delay for detection *τ* and network lifetime L to obtain the optimal design variables. The deflection coefficient curves in Figure 13 are obtained with numerical optimization using MATLAB Optimization Toolbox fmincon function along with the interior-point algorithm [25].

Monte Carlo simulations (MCS): MCS is used to simulate the statistical behavior of the wireless network example in Figure 10 for different design schemes. The MCS is used to: (1) verify the analytical and numerical results in the paper and; (2) obtain the ROC curves for the presented design approaches, which are hard to obtain using analytical techniques.. The simulation is set up as follows:

1. Network parameters: The optimal design variables obtained from the numerical optimization are used for each design approach.
2. Hypothesis: MCS is performed for both $H_{0}$ and $H_{1}$ , to evaluate the deflection coefficient and ROC curve.
3. Sensors: Observations are generated locally at each sensor for each communication slot. Each sensor attempts transmission according to the network transmission scheme.
4. Communication channel: The channel state for each sensor is simulated for each detection cycle.
5. Fusion center: The fusion center calculates the test statistic *V* and the final decision for each simulation run.
6. Performance evaluation:
  (a) Simulation runs: The number of MCS runs is 1500 for each design case.
  (b) Deflection coefficient: This is evaluated using the basic definition in Equation (17) and the simulation data.
  (c) ROC curve: This is evaluated by varying the detector threshold between ]−∞,∞[ , for each delay value.

The ROC curves in Figure 14 are obtained using MCS running in the MATLAB computing environment.

### 6.1. Cross-Layer Design Performance Surface

**Figure 11:**
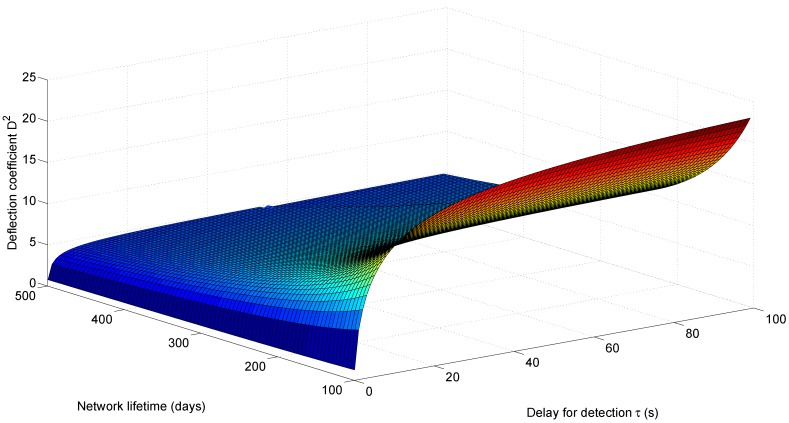
Deflection coefficient as it varies with the network lifetime and delay for detection for the slotted ALOHA with no retransmission (S-ALOHA-NR) tree sensor network.

Figure 11 shows the performance surface for the S-ALOHA-NR tree network in Figure 10, using the proposed cross-layer design approach. The surface plots the deflection coefficient for different delay and network lifetime values. For a fixed network lifetime, the deflection coefficient increases with the delay for detection, as more observations are expected at the fusion center. For a fixed delay for detection, the deflection coefficient decreases with network lifetime, as the energy budget allocated for each detection cycle decreases to prolong the network lifetime. Decreasing the energy budget reduces the probability of successful packet transmission, hence causing less observations at the fusion center.

**Figure 12:**
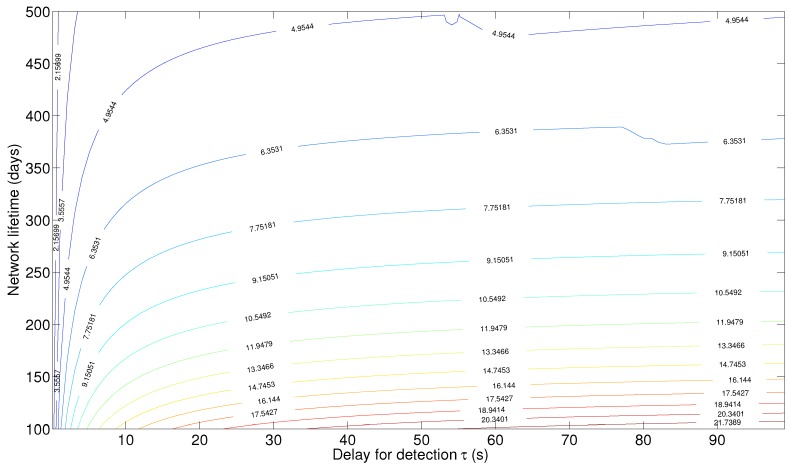
Contour plot for the deflection coefficient for the S-ALOHA-NR tree sensor network.

Figure 12 shows a contour plot for the deflection coefficient, where each curve corresponds to the set of pair values (delay for detection *τ*, network lifetime L ) that gives rise to the indicated value of the deflection coefficient. To keep the deflection coefficient constant while increasing the network lifetime, the delay for detection has to increase also, so that more observations could be received in each detection cycle. This compensates for the energy decrease as a result of a prolonged network lifetime.

In the following discussion, we resort to one-dimensional plots to compare between the different design approaches.

### 6.2. Frequency Division Multiple Access Design

In this design, each sensor has its own communication channel to the relay node or the fusion center. The communication channels are allocated to different frequency ranges and therefore orthogonal to each other. The orthogonal channels assumption has been used in the early development of decentralized detection algorithms, as it abstracts away the complexity of the communication network [1]. The orthogonal channel assumption is not always practical due to either the availability of different frequency bands or the resulting complexity in the sensor design.

The deflection coefficient and optimization problem are identical to the cross-layer case, except that the MAC contention terms in the total probability of successful packet transmission are omitted:

(60) $$\begin{array}{cc} \lambda_{k} & =\Phi \left[\left(\frac{1}{\sigma_{c}^{k}}\right)\left(10\log\frac{p_{k}-\alpha }{N_{0}W\left(2^{\frac{{\bar{R}}_{k}}{W}}-1\right)}-\mu_{c}^{k}\right)\right] \end{array}$$

**Figure 13:**
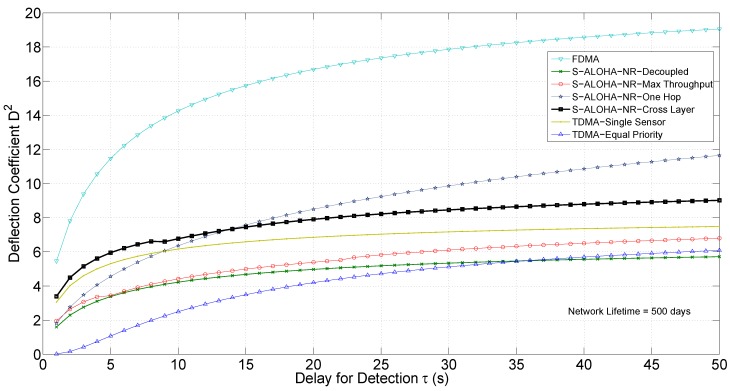
Deflection coefficient for various network designs.

**Figure 14:**
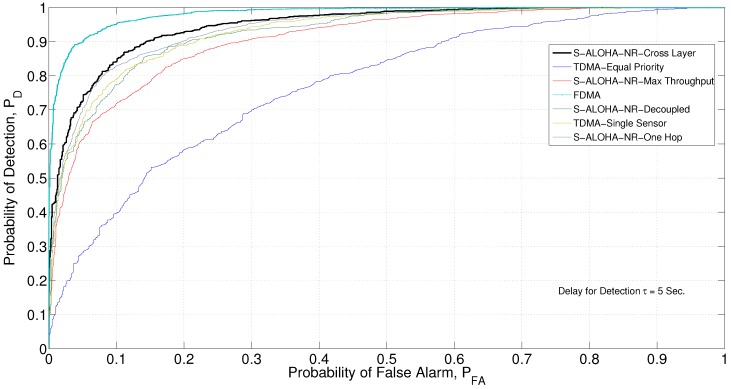
ROC curve for various network designs, Monte Carlo simulation (MCS) generated.

The orthogonal channel assumption eliminates the contention problem in the MAC network. In addition, it allows each sensor to transmit in each time slot during the detection cycle, leaving the physical channel impairment as the only source of imperfect communication. Therefore, this design scheme represents an upper bound for the given network performance. Figure 13 and Figure 14 show the deflection coefficient and the ROC curve, respectively, for the FDMA design for the example network in Figure 10.

### 6.3. Time Division Multiple Access, Equal Priority Design

In this scheme, transmissions are scheduled *a priori* in a round-robin fashion, where only one sensor transmits during any time slot for each shared communication channel. This scheme eliminates collisions between sensor nodes, and sensor transmissions are subject to channel impairments only. The deflection coefficient could be expressed as:

(61) $$\begin{array}{ll} D^{2} & =\frac{\tau }{b}J_{FC}\\ J_{k} & =\sum_{v\in C_{k}}\lambda_{v}\left[R_{v}\left(c_{v}-c_{k}\right)+J_{v}\right]\;,k=1,2,\ldots \text{FC}\\ J_{v} & =0\;\mathrm{if}\;k\;\mathrm{is}\;a\;\mathrm{leaf}\;\mathrm{node},c_{FC}=0\\ \lambda_{k} & =\Phi \left[\left(\frac{1}{\sigma_{c}^{k}}\right)\left(10\log\frac{|c_{k}|e_{k}^{r}/\tau -\alpha }{N_{0}W\left(2^{\frac{{\bar{R}}_{k}}{W}}-1\right)}-\mu_{c}^{k}\right)\right] \end{array}$$

We note that the reporting energy for each sensor is multiplied by the number of sensors sharing the communication channel, as the sensor is transmitting for only a fraction of the detection cycle.

This scheme has a degraded performance from the cross-layer design, as illustrated in Figure 13 and Figure 14, mainly because it is not selective, *i.e.*, each sensor has equal priority of transmission regardless of its QoI or CSI. This selectivity property is built in the cross-layer design approach, as the solution of the optimization algorithm selects the best sensors to transmit. The performance gap is network-dependent, and TDMA global optimization could outperform for specific network configurations. In addition, non equal-priority TDMA, where scheduling could be made between a subset of sensors that may transmit an unequal number of times, is of particular interest for further research. Non equal priority TDMA is hard to optimize, though, as the resulting problem is an integer programming problem that may be difficult to solve in real time.

### 6.4. Time Division Multiple Access, Single Sensor Transmission

Under this scheme, collisions are eliminated by selecting one branch only of the tree to transmit. The choice could be based on the leaf node QoI, *i.e.*, the sensor with the best QoI, or the leaf node CSI, *i.e.*, the sensor with the best channel. This scheme considers a single attribute only of the tree network, while the proposed cross-layer approach integrates the three sensor attributes in a formal way. Therefore, it can be shown that this scheme is always upper-bounded by the cross-layer design. The upper bound is achieved when the combined QoI, CSI and REI of the selected sensor outperforms all other combinations of sensor transmissions. In such a case, the proposed optimization algorithm will result in this transmission scheme by setting the transmission probability of all nodes connected to the selected branch to q=1 and to q=0 for all other nodes.

The deflection coefficient and optimization problem are identical to the cross-layer case, with all terms for non-transmitting sensors omitted and contention terms in the transmission probability removed. As an example, for the S5−>S8−>FC transmission:

(62) $$\begin{array}{c} D^{2}=\left(\frac{\tau }{b}\right)\lambda_{8}\left[{\bar{R}}_{8}c_{8}+\lambda_{2}{\bar{R}}_{2}\left(c_{2}-c_{8}\right)\right] \end{array}$$

The main advantage of this scheme is its simplicity, as the resulting optimization problem is much simpler with very few decision variables. Therefore, the optimization problem could be solved in real time and more frequently as the environment changes. Figure 13 and Figure 14 show the deflection coefficient and the ROC curve, respectively, for the best QoI scheme for the example network in Figure 10, where the branch S5−>S8−>FC is selected. For the given network, S5 has the best QoI, while suffering from the highest mean path loss. In addition, the energy per detection cycle is not the highest among all other sensors. This results in a degraded performance from the proposed cross-layer design.

### 6.5. S-ALOHA-NR, Single-Hop Network

Under this transmission scheme, all sensors transmit directly to the fusion center, contending on one MAC channel using the S-ALOHA-NR protocol [14]. As leaf and intermediate sensor nodes transmit a longer distance, the communication drop rate is higher. Figure 13 shows the deflection coefficient for this transmission scheme. Compared to the cross-layer tree network, the single-hop network outperforms for large delay values. The reason is that as the delay increases, the energy allocated for each transmission decreases and the channel drop rate increases. As the tree network has more than one hop, transmissions suffer from more drops than the single hop network.

The deflection coefficient for the single-hop network could be expressed as a special case of the tree network with depth = 1:

(63) $$\begin{array}{c} D^{2}=\frac{\tau }{b}\sum_{v\in C_{FC}}\lambda_{v}{\bar{R}}_{v}c_{v} \end{array}$$

Several factors affect the cut-off delay value, including the number of sensors, the transmission distances (affecting mean path loss), the number of hops in the tree network, and the energy allocated for transmission. For the given example network, doubling the transmission energy moves the cut-off delay value from 7 to 15 s. A separate comparative study between the single-hop and tree networks that investigates the impact of all design parameters is of particular interest. Figure 14 shows the ROC curve for the single-hop network for τ=5 s, where the tree network design outperforms.

### 6.6. Decoupled Design

In this approach, each layer is designed separately. In the conventional slotted ALOHA, the MAC sublayer is designed to minimize the probability of collision, without regard to the QoI and CSI for each node [26]. For the sub-tree composed of node *k* and the set of its immediate child nodes, $C_{k}$ , the minimum probability of collision occurs at $q_{v}=1/N_{k}$ , where $N_{k}=\left|C_{k}\right|$ and, consequently, $P_{t}^{k}=\left(p_{k}-\alpha \right)/N_{k}$ . The physical layer is designed to guarantee a minimum probability of successful packet transmission, $\lambda_{v}$ [27]. Using Equation (3), we obtain:

(64) $$\begin{array}{c} {\bar{R}}_{i}=W\log_{2}\left(1+10^{\left[0.1\sigma_{c}^{i}\left(a_{i}-\Phi^{-1}\left[\lambda_{v}\right]\right)+\log N_{k}\right]}\right) \end{array}$$

and using Equation (21), the deflection coefficient is given by:

D2=τbJFC(65)Jk=λvNk1−1NkNk−1∑v∈CkJv+(cv−ck)R¯i

To make a fair comparison, we do not assume a pre-set value of $\lambda_{k}$ . Rather, we optimize $\lambda_{k}$ values to yield the maximum deflection coefficient. Therefore, the local optimization problem could be written in the form:

maxλvλvNk1−1NkNk−1∑v∈CkR¯v(cv−ck)+Jv(66)   s.t.R¯v≥∑u∈CvλuR¯u=rv

where ${\bar{R}}_{v}$ is given by Equation (64).

Figure 13 and Figure 14 show the deflection coefficient and ROC curve, respectively. The decoupled design approach has a significantly degraded performance when compared to the cross-layer approach, as it does not take into account the application layer, in addition to the decoupling between the physical and MAC layers.

### 6.7. Max Throughput Design

In this approach, the performance metric of interest in designing the communication network is the throughput [28,29]. For any relay node *k* for the given tree network, the throughput could be defined as [9]

(67) $$\begin{array}{cc} T_{k} & =\sum_{v\in C_{k}}\lambda_{v}{\bar{R}}_{v}=\sum_{v\in C_{k}}{\bar{R}}_{v}q_{v}\prod_{i\in B_{v}}\left(1-q_{i}\right)\Phi \left[a_{v}-\left(\frac{10}{\sigma_{c}^{v}}\right)\log q_{v}\left(2^{\frac{{\bar{R}}_{v}}{W}}-1\right)\right] \end{array}$$

The objective is to choose the design variables $q_{v}$ and $R_{v}$ to locally maximize the throughput. The constraint on the communication rate of node *v* could be expressed in terms of its throughput as ${\bar{R}}_{v}\geq T_{v}$ , where $T_{v}=0$ for leaf nodes. The optimization problem could be formulated as:

maxqv,R¯vTk(68)   s.t.0≤qv≤1,R¯v≥∑u∈CvλuR¯u

where $\lambda_{u}$ and ${\bar{R}}_{u}$ are obtained from solving the local optimization problems at the lower level for each node *v*, as indicated in Figure 8. The optimal design variables could then be substituted back in Equation (20) to evaluate the deflection coefficient.

Figure 13 and Figure 14 show the deflection coefficient and ROC curve, respectively. The cross-layer design outperforms the max throughput by integrating the QoI into the design process.

### 6.8. Discussion

The cross-layer design approach for the tree network with the S-ALOHA-NR MAC protocol outperforms the TDMA with equal priority, TDMA with a single sensor, max throughput, decoupled, and a single-hop S-ALOHA-NR network (for small delay values). For non-real-time applications that could support large delay values, the single hop network may perform better depending on the network setup. The FDMA transmission scheme outperforms all other schemes, including the cross-layer approach, provided that the network resources are available and the sensor nodes have the frequency tuning capability.

It should be highlighted that the performance enhancement for the cross-layer approach comes with no additional hardware complexity in the network. However, the computational intensity to obtain the optimization problem solution grows with the network size. The local optimization solution presented is a suboptimal solution. However, for some sensor networks, simplicity with other schemes, e.g., TDMA with a single sensor, may be preferred despite the partial loss of detection performance.

### 6.9. Direct Transmission *versus* In-Network Processing

**Figure 15:**
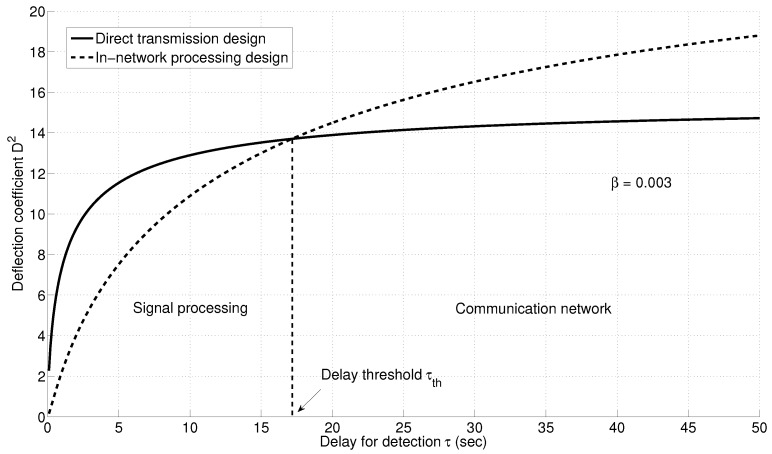
Deflection coefficient for direct transmission and in-network processing designs.

Figure 15 compares the deflection coefficient for the direct transmission and in-network processing designs for different delays for detection values for the example network in Figure 10. We note that the direct transmission design outperforms the design with local quantization for all delay for detection values below a threshold value $\tau_{th}$ . Increasing the delay for detection further causes the in-network processing design to outperform. The first region, *i.e.*, $\tau <\tau_{\text{th}}$ is where the signal processing aspect of the system dominates. In this region, the loss due to quantization cannot be compensated, since the delay allowed is small and not enough measurements can be collected to compensate for the quantization loss. The direct transmission scheme outperforms, since information is transmitted without prior processing. In addition, a shorter delay allows the reporting energy to be concentrated over a smaller duration, resulting in higher power for each sensor. The high power mitigates the channel impairments, and therefore, the communication network aspect is not dominant. The situation is reversed when $\tau >\tau_{\text{th}}$ . In this region, energy is distributed over a longer period, which results in low sensor transmission power, and hence, the channel impairment is dominant in determining the system performance. The in-network processing design requires a lower communication rate, thereby mitigating the channel impairment. On the other hand, the direct transmission design requires a higher communication rate, resulting in packet loss and degraded system performance.

**Figure 16:**
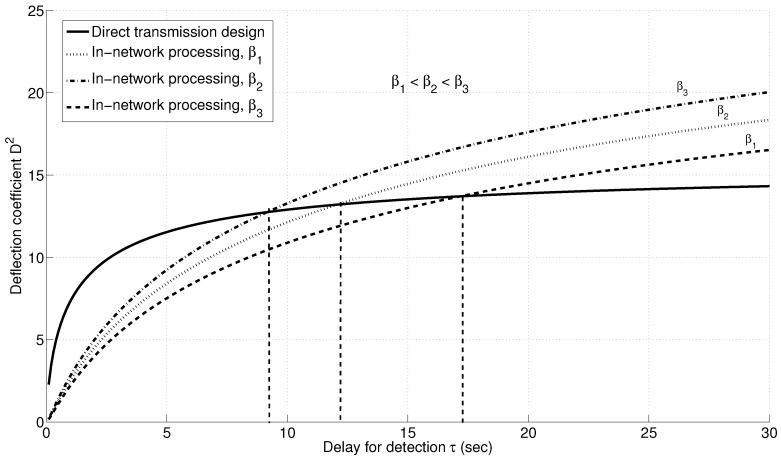
Variation of the detection threshold with quantizer design parameter β.Variation of detection threshold with quantizer design parameter

The delay threshold depends mainly on the quantizer design, summarized by the parameter β, in addition to the signal to noise ratio for each sensor and the energy allocated for the detection process. Figure 16 shows the variation in the delay threshold with the quantizer design parameter β. As β increases, the exponential decay rate for the quantization effect is much faster; hence, the threshold is lower.

## 7. Conclusions

In this paper, we used a model-based approach to design a tree-structured, slotted ALOHA with no retransmission sensor network, for detection applications. We developed an integrated model for the detection system and integrated the QoI, REI and CSI quality measures into the design process. We designed the communication rate and transmission probability for each tree node. The proposed model-based approach shows a performance gain over decoupled and maximum throughput contention-based networks, as well as equal priority and single-sensor TDMA contentionless networks. This performance enhancement comes with no additional network sources.

The choice between single-hop and tree networks depends on several factors, including the number of sensors, the distance between sensors and the fusion center, the tree formation and the number of hops, as well as the energy allocated for transmission. For the presented example network, the tree network outperforms for small delay values, while the single-hop network results in better detection performance for large delay values. A more in-depth comparative analysis between the two networks and the effect of various network parameters is of particular interest.

For applications with stringent delay requirements, we show that system design with direct transmission of sensor observations results in better performance since the channel impairment is unlikely to play a major role. If the application can tolerate longer delays, then the design with in-network processing results in better detection performance, as the communication network becomes a dominant factor in determining the system performance.

Despite the fact that the cross-layer design approach results in a no-cost performance increase, this approach has its own pitfalls. First, the mathematical model that captures the inter-relationships between different layers is in general complex, as evidenced by the model in this paper. Because of this complexity, several iterations through the design process have to be made in order to obtain a tractable model. Often times, simplifying assumptions are required, and it is not always straightforward to check the validity of such assumptions. Second, the optimization problem obtained has to be solvable in real time with existing optimization algorithms. This is not always possible, as the optimization problem complexity is closely coupled to the model complexity. Despite these pitfalls, the cross-layer design complexity is justified when it is desired to optimize the performance with limited system resources that cannot be replenished (e.g., remote WSN in a battlefield). The decoupled and TDMA schemes, on the other hand, may be justified for systems with enough resources, such that the performance loss could be compensated by additional resource allocation.

We assumed that the energy is pre-allocated to each sensor based on its energy reserve. Optimal energy allocation to maximize the detection performance is one possible extension to the presented work. In this case, care should be taken that the energy of relay nodes is not depleted before its descendants. Finally, we assumed fixed allocation schemes in this paper, where the transmission pattern and network parameters are calculated *a priori*. Dynamic allocation schemes that cope with environmental changes are expected to outperform despite their complexity. The non-equal priority TDMA transmission scheme is one example that needs to be investigated.

## A. Proof of Proposition 1

**Proof.** At the fusion center, the LLR is an optimal test. Using the independence assumption of sensor observations, the LLR could be expressed as a sum of the individual sensor LLRs provided that the information is transmitted successfully to the fusion center. The information transmitted by sensor $v_{l}$ at tree depth *l* and time slot $i_{l}$ will be received at the fusion center if all transmissions in the slot sequence $i_{1}\ldots i_{l}$ were successful, *i.e.*, $r_{v_{1}}\left[i_{1}\right]r_{v_{2}}\left[i_{1}i_{2}\right]\ldots r_{v_{l}}\left[i_{1}\ldots i_{l}\right]=1$ . Using this fact, the LLR test could be expressed as:

 ∑i1=1L1∑v1∈Cfrv1[i1]Lxv1[i1]+∑i1=1L1∑v1∈Cf∑i2=1m1∑v2∈Cv1rv1[i1]rv2[i1i2]Lxv2[i1i2]+(69)…+∑i1=1L1∑v1∈Cf…∑il=1ml−1∑vl∈Cvl−1rv1[i1]…rvl[i1…il]Lxvl[i1…il]H1≷H0lnγ

where the LLR could be expressed as:

(70)
$$\begin{array}{c} L\left(x_{v_{l}}\left[i_{1}\ldots i_{l}\right]\right)=\frac{\mu^{v_{l}}}{\sigma_{s}^{{v_{l}}^{2}}}\sum_{j_{l}=1}^{n_{v_{l}}}x_{v_{l}}\left[j_{l},i_{1}\ldots i_{l}\right]-\frac{1}{2}n_{v_{l}}{\left(\frac{\mu^{v_{l}}}{\sigma_{s}^{v_{l}}}\right)}^{2} \end{array}$$

Using Equation (70) in Equation (69) and factoring out, the LLR test is then given by:

V=∑i1=1L1∑v1∈Cf∑j1=1nv1rv1[i1]μv1σsv12xv1[j1,i1]+∑i2=1m1∑v2∈Cv1∑j2=1nv2rv2[i1i2]μv2σsv22xv2[j2,i1i2]+…+∑il=1ml−1∑vl∈Cvl−1∑jl=1nvlrvl[i1…il]μvlσsvl2xvl[jl,ili2…il]…H1≷H012∑i1=1L1∑v1∈Cfrv1[i1]nv1μv1σsv12+∑i2=1m1∑v2∈Cv1rv2[i1i2]nv2μv2σsv22+(71)…+∑il=1ml−1∑vl∈Cvl−1rvl[i1…il]nvlμvlσsvl2…+lnγ

□

## B. Proof of Proposition 2

**Proof.** We use the definition of the deflection coefficient in Equation (17). We use the fact that $r_{i}\left[k\right]$ and $x_{i}\left[j,k\right]$ are both i.i.d. and independent. In addition, $r_{v_{i}}$ and $r_{v_{j}}$ are independent for i≠j and $E\left[r_{v_{i}}\right]=\lambda_{v_{i}}$ :

E[V;H0]=∑i1=1L1∑v1∈Cf∑j1=1nv1Erv1[i1]μv1σsv12Exv1[j1,i1];H0+(72)…+∑il=1ml−1∑vl∈Cvl−1∑jl=1nvlErvl[i1…il]μvlσsvl2Exvl[jl,ili2…il];H0=0

E[V;H1]=∑i1=1L1∑v1∈Cf∑j1=1nv1Erv1[i1]μv1σsv12Exv1[j1,i1];H1+…+∑il=1ml−1∑vl∈Cvl−1∑jl=1nvlErvl[i1…il]μvlσsvl2Exvl[jl,ili2…il];H1(73)=L1∑v1∈Cfλv1nv1μv1σsv12+m1∑v2∈Cv1λv2nv2μv2σsv22+…ml∑vl∈Cvlλvlnvlμvlσsvl2…

To find $\text{var}[V;H_{0}]$ , we expand the test statistic, noting that transmissions in different slots are i.i.d.:

var[V;H0]=L1var∑v1∈Cf∑j1=1nv1rv1[i1]μv1σsv12xv1[j1,i1]+L1m1∑v1∈Cf∑v2∈Cv1∑j1=1nv1∑j2=1nv2rv1[i1]rv2[i1i2]μv2σsv22xv2[j2,i1i2]+…(74)+∑i1=1L1∑v1∈Cf…∑il=1ml−1∑vl∈Cvl−1rv1[i1]…rvl[i1…il]μvlσsvl2xvl[jl,i1…il]

We use the identity:

(75)
$$\begin{array}{c} \text{var}\left(\sum_{i=1}^{N}X_{i}\right)=\sum_{i=1}^{N}\sum_{j=1}^{N}\text{COV}\left(X_{i},X_{j}\right)=\sum_{i=1}^{N}\sum_{j=1}^{N}E\left[X_{i}X_{j}\right]-E\left[X_{i}\right]E\left[X_{j}\right] \end{array}$$

The second term in Equation (75) vanishes for all terms in Equation (74) since the observations have zero mean under $H_{0}$ . In addition, the first term vanishes except when i=j . Accordingly:

var[V;H0]=L1∑v1∈Cfnv1E[(rv1)2]E[(xv1)2;H0]μv1σsv122+L1m1∑v1∈Cf∑v2∈Cv1nv2E[(rv1)2]E[(rv2)2]E[(xv2)2;H0]μv2σsv222+…(76)+L1m1…ml∑v1∈Cf…∑vl∈Cvl−1E[(rv1)2]…E[(rvl)2]E[(xvl)2;H0]μvlσsvl22

We note that $E\left[{\left(r_{v}\right)}^{2}\right]=\lambda_{v}$ and $E\left[{\left(x_{v}\right)}^{2}\right]=\sigma_{s}^{v^{2}}$ . Accordingly:

var[V;H0]=L1∑v1∈Cfnv1λv1μv1σsv12+L1m1∑v1∈Cf∑v2∈Cv1nv2λv1λv2μv2σsv22+…(77)+L1m1…ml∑v1∈Cf…∑vl∈Cvl−1λv1…λv1μvlσsvl2

and factoring out, we get:

(78)
$$\begin{array}{c} \text{var}\left[V;H_{0}\right]=L_{1}\sum_{v_{1}\in C_{f}}\lambda_{v_{1}}\left[n_{v_{1}}{\left(\frac{\mu^{v_{1}}}{\sigma_{s}^{v_{1}}}\right)}^{2}+m_{1}\sum_{v_{2}\in C_{v_{1}}}\lambda_{v_{2}}\left[n_{v_{2}}{\left(\frac{\mu^{v_{2}}}{\sigma_{s}^{v_{2}}}\right)}^{2}+\ldots m_{l}\sum_{v_{l}\in C_{v_{l}}}\lambda_{v_{l}}n_{v_{l}}{\left(\frac{\mu^{v_{l}}}{\sigma_{s}^{v_{l}}}\right)}^{2}\right]\ldots \right] \end{array}$$

From Equations (17), (72), (73) and (78) and by defining $c_{v}={\left(\mu^{v}/\sigma_{s}^{v}\right)}^{2}$ , we obtain Equation (20) □

## C. Proof of Proposition 3

**Proof.** First we obtain the statistics of the quantized random variable *Y*. From Equations (8) and (23):

(79)
$$\begin{array}{c} E\left[Y\right]=\Delta \left(E\left[\left\lfloor \frac{Z}{\Delta }\right\rfloor \right]+\frac{1}{2}\right) \end{array}$$

and using the series expansion of the floor function:

(80)
$$\begin{array}{c} \left\lfloor x\right\rfloor =x-\frac{1}{2}+\frac{1}{\pi }\sum_{k=1}^{\infty }\frac{\sin(2\pi kx)}{k} \end{array}$$

we get:

(81)
$$\begin{array}{c} E\left[Y\right]=E\left[Z\right]+\frac{\Delta }{\pi }\sum_{k=1}^{\infty }\frac{1}{k}E\left[\sin\left(\frac{2\pi k}{\Delta }Z\right)\right] \end{array}$$

Denote $\theta_{k}=2\pi k/\Delta$ , and using the expansion $\sin\left(x\right)=\left(e^{jx}-e^{-jx}\right)/2$ to find the expectation for the second term:

E[Y]=μZ+Δπ∑k=1∞1k12jEejθkZ−Ee−jθkZ(82)=μZ+Δπ∑k=1∞1k12jejθkμzEejθkZ−μz−e−jθkμzEe−jθkZ−μz

Using the exponential function expansion and noting that the odd central moments for the Gaussian distribution are equal to zero, we obtain:

E[Y]=μZ+Δπ∑k=1∞1ksinθkμZe−θk2σZ2(83)=μZ+Δπ∑k=1∞Δksin2πkΔμZe−2πkΔ2σZ2

and using the distribution of *Z* in Equation (7), we obtain:

(84)E[Y;H0]=0E[Y;H1]=nμσs2+Δπ∑k=1∞1ksin2πknΔμσs2e−2πkΔ2nμσs2(85)=nμσs2+δ

For the variance:

var[Y;H0]=E[Y2;H0]=Δ2EzΔ+122(86)=σZ2+Δπ2E∑k=1∞1ksin2πkΔZ2+2ΔπEZ∑k=1∞1ksin2πkΔZ

Similar to the derivation for the expected value, the last term vanishes, since all moments are odd. Therefore:

var[Y;H0]=σZ2+Δπ2E∑k1=1∞∑k2=1∞1k1k2sin2πk1ΔZsin2πk2ΔZ(87)=σZ2+Δπ2E∑k1=1∞∑k2=1∞1k1k2cos2π(k1−k2)ΔZ−cos2π(k1+k2)ΔZ

Similar to the expected value derivation, we use $\cos x=(e^{jx}-e^{-jx})/2$ , and after some algebraic manipulations, we obtain:

var[Y;H0]=nμσs2+Δπ2∑k1=1∞∑k2=1∞12k1k2cos2π(k1−k2)nΔμσs2e−2π(k1−k2)Δ2nμσs2−cos2π(k1+k2)nΔμσs2e−2π(k1+k2)Δ2nμσs2(88)=nμσs2+δ′

Similar to the derivation of Proposition (2), we take the expectation and variance of Equation (22), and using Equations (84), (85) and (88), we obtain:

(89)
$$\begin{array}{l} D^{2}=\frac{{\left(L_{1}\sum_{v_{1}\in C_{f}}\lambda_{v_{1}}\left[n_{v_{1}}{\left(\frac{\mu^{v_{1}}}{\sigma_{s}^{v_{1}}}\right)}^{2}+\delta_{v_{1}}+m_{1}\sum_{v_{2}\in C_{v_{1}}}\lambda_{v_{2}}\left[n_{v_{2}}{\left(\frac{\mu^{v_{2}}}{\sigma_{s}^{v_{2}}}\right)}^{2}+\delta_{v_{2}}\ldots m_{l}\sum_{v_{l}\in C_{v_{l}}}\lambda_{v_{l}}n_{v_{l}}{\left(\frac{\mu^{v_{l}}}{\sigma_{s}^{v_{l}}}\right)}^{2}+\delta_{v_{l}}\right]\ldots \right]\right)}^{2}}{L_{1}\sum_{v_{1}\in C_{f}}\lambda_{v_{1}}\left[n_{v_{1}}{\left(\frac{\mu^{v_{1}}}{\sigma_{s}^{v_{1}}}\right)}^{2}+\delta_{v_{1}}^{\prime }+m_{1}\sum_{v_{2}\in C_{v_{1}}}\lambda_{v_{2}}\left[n_{v_{2}}{\left(\frac{\mu^{v_{2}}}{\sigma_{s}^{v_{2}}}\right)}^{2}+\delta_{v_{2}}^{\prime }+\ldots m_{l}\sum_{v_{l}\in C_{v_{l}}}\lambda_{v_{l}}n_{v_{l}}{\left(\frac{\mu^{v_{l}}}{\sigma_{s}^{v_{l}}}\right)}^{2}+\delta_{v_{l}}^{\prime }\right]\ldots \right]} \end{array}$$

□

## D. Proof of Proposition 4

**Proof.** Single sensor transmission solution: From the stationarity condition in Equation (40), it could be easily shown by direct substitution, that for silent sensors, $q_{i}=R_{i}=0$ satisfies all KKT conditions. Therefore, this point is a candidate for a local maximizer. For the only active sensor that transmits with probability one, *i.e.*, $q_{\nu }=1$ , if $q_{i}\neq 0$ for any other sensor i≠ν , then a collision is guaranteed when sensor *i* attempts transmission. Therefore, the parent node will not receive any information from sensor *i*. Clearly, $q_{i}$ should be set to zero ∀i≠ν , *i.e.*, all other sensors have to be silent. The same result could also be obtained by direct inspection of the objective function, when $q_{\nu }=1$ , where any value of $q_{i}\neq 0$ will cause the objective function value to decrease. Therefore, $q_{i}=0$ . We conclude that we have a set of $|C_{k}|$ candidate points, ( $q_{\nu }=1,q_{i}=0,i\neq \nu$ ), for a local maximum.

Stationary point solution: For the active sensors, $0<q_{i}<1$ , and therefore, all constraints are inactive. Accordingly, all Lagrange multipliers are =0 , *i.e.*, ν=0 , and from the stationarity condition, the optimal values for q and R are equal to the stationary point $x^{*}$ , where $\nabla J(x^{*})=0$ . □
